# Early life adversity jointly regulates body-mass index and working memory development

**DOI:** 10.1098/rspb.2023.1945

**Published:** 2023-11-15

**Authors:** Bence Csaba Farkas, Pierre Olivier Jacquet

**Affiliations:** ^1^ Université Paris-Saclay, UVSQ, Inserm, CESP, 94807, Villejuif, France; ^2^ Institut du Psychotraumatisme de l'Enfant et de l'Adolescent, Conseil Départemental Yvelines et Hauts-de-Seine et Centre Hospitalier des Versailles, 78000, Versailles, France; ^3^ Centre de recherche en épidémiologie et en santé des populations, Inserm U1018, Université Paris-Saclay, Université Versailles Saint-Quentin, Paris, France; ^4^ LNC2, Département d’études Cognitives, École Normale Supérieure, INSERM, PSL Research University, 75005, Paris, France

**Keywords:** neurocognitive development, somatic development, early life adversity, human life history strategies, sex differences, structural equation modelling

## Abstract

Previous work has proposed that balancing energy expenditure towards body and brain development in an optimal fashion results in a negative relationship between somatic and neurocognitive growth during development. An important issue, largely overlooked so far, is the extent to which this energetic trade-off is influenced by early life environmental factors. In this study, we estimated the association between neurocognitive (measured by working memory ability) and somatic (measured by body-mass index) developmental trajectories, while taking into account multiple dimensions of early life adversity. Results of our initial growth curve model were consistent with this brain–body trade-off in both girls and boys. In a subsequent model, we showed that early life adversity had positive associations with somatic and negative associations with neurocognitive growth trajectories, although the direct negative coupling between them remained consistent. Finally, a multidimensional adversity model, separating the effects of deprivation, threat and unpredictability, revealed that the dimension of deprivation—reflecting lack of access to resources and cognitive stimulation—contributed the most to both somatic and neurocognitive growth patterns. These results suggest that the way individuals balance energy between these two biological constructs during development is partly linked to environmental influences through phenotypic plasticity.

## 1. Introduction

The human brain is an energetically costly organ that requires about 20% of resting energy expenditure, despite representing only about 2% of body weight [1,2]. Its energetic requirements are even higher during childhood, with recent studies estimating that at its lifetime peak energetic requirement around 4–5 years of age, the brain accounts for roughly 66% of resting energy expenditure [1]. This intense and variable energetic demand is due to the developmental pattern of synaptic connections, that is characterized by a period of overproduction during early childhood, followed by an extended period of experience-dependent pruning [3,4]. As the brain lacks any ability to store energy itself, it must compete and coordinate with the body for metabolic substrate [5]. This has been proposed to give rise to a trade-off between somatic and brain development [6]. Evidence for such a trade-off comes from landmark studies [1,7–9] revealing a strong inverse relationship between brain metabolism and the rate of weight gain throughout childhood, with highest brain energetic demands corresponding to the lowest body weight gains.

More recently, researchers have hypothesized an indirect expression of this trade-off through its consequences on cognitive development, given that a significant portion of the energy allocated to the brain is devoted to the extended maturation of the prefrontal cortex that underpins high-level cognitive skills [6,7,10,11]. In this respect, a study by Blair *et al*. [12] has recently demonstrated a negative coupling between the developmental trajectories of body mass index (BMI) and executive function abilities (EF) in a longitudinal sample of 24- to 60-month-old children. Despite the indirect and thus seemingly imprecise nature of BMI and EF measures, many studies show that they can serve as proxies of energy storage and/or expenditure by the body and the brain [6,13].

Overall, these observations suggest that the way an organism ‘arbitrates’ energy allocation between somatic growth and brain maturation could be an important source of phenotypic variability and represent a relatively unexplored risk factor for obesity and related physical health issues (e.g. metabolic syndrome) [14] and for cognitive functioning [6]. The present study draws attention to a factor that, although relatively neglected so far, could nonetheless exert a substantial influence on the shape of this energy allocation pattern, i.e. the types of adversity an individual experiences early in life. Early life adverse experiences, such as exposure to maltreatment, abuse, poverty, or a lack of peer and parental support, have been consistently linked both to reduced EF abilities [15–17] and to increased BMI and obesity risk [18–20]. These findings can be interpreted in light of life-history theory, the prevailing framework in explaining variation in energy expenditure into various biological functions [21,22]. In developmental psychology, the role of early life adversity has been understood as providing cues about the levels of danger and unpredictability in future environmental states, and leading to life history strategies, characterized by investment into short timescale, reproductive goals, at the expense of long timescale goals and somatic maintenance [21,23,24]. Such a lifestyle may involve relatively increased somatic growth to reach reproductive maturity earlier, and relatively decreased growth of executive functions and health efforts which would only be associated with fitness benefits in safe and predictable environments. Thus, any negative association between EF and BMI must be interpreted in light of the possibility that it arises due to the effects of early life adversity in calibrating energy allocation decisions.

While some works investigating EF–BMI links have included adversity measures in their modelling [12,25], their operationalization was rooted in the so-called cumulative risk approach, which only considers the number of adverse experiences as predictors of developmental outcomes [26]. Recent theoretical and empirical work has instead highlighted the importance of distinguishing between different dimensions of adversity, with partially distinct effects [26–29]: (i) Threat, conceptualized as a source of harshness capturing morbidity–mortality from harm imposed by other agents; (ii) Deprivation, conceptualized as a source of harshness capturing morbidity–mortality from insufficient environmental inputs; and (iii) Unpredictability, conceptualized as spatiotemporal variability in both Threat and Deprivation. Identifying which adversity dimensions are related to somatic and neurocognitive development is therefore important in several respects. First, it might help clarify the way the organism allocates the energy between the two. Second, it might help future research better understand the emergence of the related physical and mental health costs.

In this study, we thus attempted to expand on earlier research into the EF–BMI relationship, by incorporating this more detailed dimensional model of adversity. To this end, we made use of the Early Childhood Longitudinal Study (ECLS), a large (*N* = 15 038), nationally representative, longitudinal dataset of children between the ages of 6 and 11 [30]. We constructed composite scores, reflecting children's exposure to adversity factors indexing Deprivation, Threat and Unpredictability, and related these to the growth of working memory (WM), an important component of EF, and BMI, using latent growth curve modelling [31].

## 2. Methods

### (a) Sample

Data were drawn from the ECLS, Kindergarten Class of 2010–11 (ECLS-K : 2011). The ECLS-K : 2011 is a USA, nationally representative, longitudinal cohort of children. For a detailed description of sampling procedure, refer to ch. 4 of the ECLS, Kindergarten Class of 2010–11 (ECLS-K : 2011), User's Manual for the ECLS-K : 2011 Kindergarten–Fifth Grade Data File and Electronic Codebook, Public Version (NCES 2019-051) [30] and the study website (https://nces.ed.gov/ecls/kindergarten2011.asp). Data from the autumn and spring assessment periods of all waves were accessed and used in this study, covering focal child ages 6–11. The ECLS-K : 2011 was directed by the National Center for Education Statistics (NCES) and approved for data collection by the NCES ethics review board. Parental written informed consent was obtained at study enrolment.

From the full sample of 18 174 children, those with missing values on the Birthyear, Race/Ethnicity or Sex variables, and those whose primary language was not English, were excluded. This left a sample of 17 794 children. From this, participants with missing values on more than half of our primary variables (10 out of 21) were also excluded. This left a final sample of 15 257 children. Our analyses of missing data patterns and their handling during model estimation are provided in electronic supplementary material, text S2.

### (b) Measures

We were interested in investigating how exposure to multiple dimensions of early life adversity, at the first timepoint (age 6), are related to trajectories of growth in WM and BMI between the first and last timepoints (between ages 6 and 11). To capture adversity exposure, we constructed aggregated scores, from various indicators. All scores were sums of *z*-scored indicators [21,32]. All variables were coded so that higher values are indicative of greater deprivation, threat or unpredictability. A detailed description of the indicators is provided in electronic supplementary material, text S1. Summary statistics for each adversity indicator are also reported in electronic supplementary material, table S1.

#### (i) Deprivation

The deprivation score was a sum of the *Poverty*, *Food security*, *Single parenthood*, *Food stamps*, *Parent-teacher conference*, *Parental warmth*, *Cognitive stimulation* and *Maternal depression* indicators. What ties all these indicators together is that exposure to them decreases the available resources or indicates the lack of a supportive environment for typical psychological development and learning.

#### (ii) Threat

The threat score was a sum of *Neighbourhood crime*, *School violence*, *Parental stress* and *Neighbourhood safety* indicators. What ties all these indicators together is that exposure to them confers a risk of physical or psychological harm.

#### (iii) Unpredictability

The unpredictability score was a sum of *Care arrangements*, *Jobs*, *Dinner routine*, *Household size*, *Maternal mood entropy*, *Moves*, *Partner change*, *School change* and *Household size change* indicators. What ties all these indicators together is that exposure to them reduces the predictability of day-to-day environments or signals a changepoint in the environment.

#### (iv) General adversity

We also constructed a general adversity score, reflecting the operationalization of adversity according to the cumulative risk model, which does not consider separable dimensions. This composite was simply the sum of all indicators detailed above, without separating deprivation, threat or unpredictability. The model with this composite serves as a useful baseline to which we can compare the additive value of our main dimensional model.

#### (v) Working memory

Children's WM was assessed with the Numbers Reversed subtest of the Woodcock–Johnson III Tests of Cognitive Abilities [33]. We used the W score, which is a type of standardized score, that is particularly useful for the measurement of growth. It has a mean of 500 and standard deviation of 100. Its mean has been set by the publisher to the average performance of a child of 10 years.

We used the W score of children at the spring assessment from each year of the study, corresponding to 6 data points for each child, with approximately equal yearly intervals, covering child ages 6, 7, 8, 9, 10 and 11. These scores were grand mean standardized before being entered into the models, i.e. the whole sample mean score from all assessments was subtracted from each score, and the resulting value was divided by the whole sample standard deviation from all assessments.

#### (vi) Body mass index

Composite BMI scores in the ECLS were calculated by multiplying a child's unrounded weight in pounds by 703.0696261393 and dividing by the square of the child's composite unrounded height in inches [34,35. We used the BMI score of children at thepring assessment from each year of the study corresponding to 6 data points for each child with approximately equal yearly intervals covering child ages 6 7 8 9 10 and 11X2BMI X4BMI X6BMI X7BMI X8BMI X9BMI]. These scores were also grand mean standardized before being entered into the models.

#### (vii) Covariates

*Sex*. Information about child's sex was collected from schools at the time of sampling, collected from parents in the autumn parent interview, and confirmed by parents in the spring parent interview. Sex was coded as 1 (male) or 2 (female).

*Birthyear*. The child's date of birth composite variable was derived from the parent interview and other sources if necessary to resolve discrepancies. Birthyear was coded as 1 (2003/2004) or 2 (2005/2006).

*Race/Ethnicity*. We used a race/ethnicity composite variable that draws from either the parent-reported data about the child's race or other sources if parent responses about the child's race were missing. This variable was simplified and recoded as 1 (white) or 2 (non-white) for the model.

*Physical activity*. Carers indicated on how many days in a typical week the focal child gets exercise that causes rapid breathing, perspiration, and a rapid heartbeat for 20 continuous minutes or more. This variable ranged between 0 (no days) and 7 (all days).

### (c) Statistical analysis

All analyses used ECLS-K : 2011 sampling weights that account for nonresponse and potential under-coverage of the target population in all waves. All pre-processing and statistical analysis was performed in R 4.2.1 [36]. For data visualization, the *ggplot2* package was used [37]. Additional packages used for pre-processing, visualization and analyses were *tidyverse* [38], *psych* [39] and *semTools* [40]. An alpha level of 0.05 was applied to all analyses.

We fitted a series of latent growth curve models in order to estimate the trajectories of change in WM and BMI scores [31]. After the establishment of appropriate growth curves by unconditional models, we proceeded to fit the conditional growth curve model, that simultaneously estimated growth parameters for both WM and BMI, and the regression paths between them and our covariates of Physical activity, Race/Ethnicity and Birthyear. At the final stage of the analysis, we added the early life adversity composite scores as predictors of all growth parameters. We tested both a cumulative adversity composite reflecting general adversity and dimensional composites of deprivation, threat and unpredictability. Sex differences were tested by *χ*^2^ difference testing of nested models. Following standard practice, model fit was evaluated using the comparative fit index (CFI), the root mean square error of approximation (RMSEA) and the standardized root mean square residual (SRMR). According to a commonly used guideline, CFI values higher than 0.95, RMSEA values lower than 0.05 and SRMR values lower than 0.08 are generally considered indicators of good model fit [41]. We accounted for the complex survey design using the *lavaan.survey* package [42]. More details about the modelling are provided in electronic supplementary material, text S3.

## 3. Results

Demographic information of the sample is presented in table 1; summary statistics and fraction of missing values for primary variables and bivariate correlations between them are presented in electronic supplementary material, tables S4–S6. Distributions of primary variables are presented in electronic supplementary material, figure S1. Naturally, there were high positive correlations between the different adversity variables, and between the WM and BMI scores at different timepoints. Adversity composites tended to correlate negatively with WM, and positively with BMI. Overall, WM and BMI indicators tended to show negative correlations. Physical activity was positively correlated with WM, negatively with BMI and with the adversity composites. The number and percentage of participants considered obese, overweight, normal weight, and underweight at each timepoint according to CDC reference data are also reported in electronic supplementary material, table S7. Most of the sample was in the normal weight range in all timepoints (approx. 60–70%), with the rest of the sample falling into either the overweight or obese categories (approx. 15–20% each). Extremely few individuals fell into the underweight range (approx. 3%). Rates of overweight and obese status also notably increase during the studied period, entirely in line with previous estimates, from both the USA [43] and Europe [44].

**Table 1.** Demographic information of the sample. For categorical variables, numerical values are unweighted counts and percentages in parentheses are weighted proportions. For continuous variables, numerical values are weighted means and numerical values in parentheses are weighted standard errors of the mean. Comparisons are based on a Kruskal–Wallis test for continuous data and a *χ*^2^ test with Rao–Scott second-order correction for discrete data.

variable	unweighted N (weighted %) or weighted M (weighted SE)		*p*-value
sex	male	female	
	7809 (51.44%)	7448 (48.56%)	—
age at first assessment (months)	67.84 (0.09)	67.20 (0.09)	< 0.001
race/ethnicity			0.179
white, non-Hispanic	3816 (52.47%)	3553 (50.88%)	
Black/African-American, non-Hispanic	926 (13.38%)	857 (13.30%)	
Hispanic, race specified	1912 (24.26%)	1832 (24.82%)	
Hispanic, no race specified	90 (0.41%)	83 (0.20%)	
Asian, non-Hispanic	599 (3.74%)	676 (5.23%)	
Native Hawaiian/Pacific islander, non-Hispanic	44 (0.43%)	41 (0.35%)	
American Indian/Alaska native, non-Hispanic	63 (1.26%)	64 (1.15%)	
two or more races, non-Hispanic	359 (4.05%)	342 (4.07%)	
education level of primary caregiver			0.642
8th grade or below	361 (4.33%)	318 (4.92%)	
9th–12th grade	613 (8.24%)	560 (7.52%)	
high school diploma/equivalent	1535 (23.51%)	1473 (23.58%)	
vocational/technical programme	370 (6.09%)	397 (6.82%)	
some college	1908 (27.91%)	1729 (26.47%)	
bachelor's degree	1439 (18.26%)	1422 (18.52%)	
graduate/professional school—no degree	132 (1.68%)	115 (1.46%)	
master's degree or higher	818 (9.73%)	781 (10.33%)	
missing data	633 (0.26%)	653 (0.38%)	
household income (yearly)			0.288
$5000 or less	198 (3.00%)	168 (2.28%)	
$5001 to $10 000	256 (3.55%)	239 (2.88%)	
$10 001 to $15 000	365 (4.36%)	381 (5.58%)	
$15 001 to $20 000	409 (5.55%)	389 (5.00%)	
$20 001 to $25 000	506 (7.30%)	442 (6.33%)	
$25 001 to $30 000	317 (4.74%)	314 (5.37%)	
$30 001 to $35 000	327 (4.46%)	281 (4.84%)	
$35 001 to $40 000	290 (4.11%)	295 (4.47%)	
$40 001 to $45 000	220 (2.82%)	202 (3.08%)	
$45 001 to $50 000	243 (3.77%)	213 (2.99%)	
$50 001 to $55 000	211 (3.30%)	187 (3.06%)	
$55 001 to $60 000	197 (2.70%)	196 (3.15%)	
$60 001 to $65 000	214 (3.24%)	193 (2.79%)	
$65 001 to $70 000	170 (2.97%)	192 (2.88%)	
$70 001 to $75 000	250 (3.45%)	233 (3.35%)	
$75 001 to $100 000	853 (11.31%)	817 (11.47%)	
$100 001 to $200 000	1082 (12.73%)	1030 (13.94%)	
$200 001 or more	296 (3.85%)	260 (3.25%)	
missing data	1405 (12.80%)	1416 (13.29%)	

### (a) Unconditional growth curves

Latent growth curve models were used to test our main hypotheses concerning the association between BMI and WM growth (interpreted as proxies of energy expenditure on brain, and somatic development, respectively) modelled from the six data points, approximately equally spaced between the respondents' 6th and 11th years of life.

Model selection results reported in electronic supplementary material, table S8, showed that the WM growth and BMI growth were best captured by the models which included both linear and quadratic components. The quadratic growth curve model was therefore employed in subsequent analyses. For the sake of brevity and clarity of reading, we do not detail the effects of the adjustment variables. The full list of model parameters is available in the electronic supplementary material in the form of .Rda files.

### (b) Conditional growth curves: baseline model

After the establishment of appropriate growth curves, we proceeded to fit the conditional growth curve model, that simultaneously estimated growth parameters for both WM and BMI (adjusted for Physical activity, Race/Ethnicity and Birthyear) and the regression paths between them. As both the growth curves and the associations between the WM and BMI growth parameters could show sex specificity, we also tested the equality of this model across sexes.

#### (i) Goodness of fit

**Figure 1.**
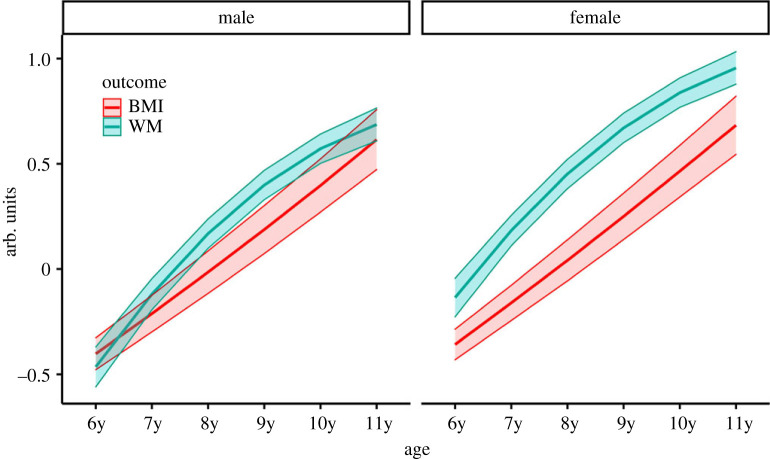
Model-implied average trajectories of BMI (in red) and WM (in blue). Results are separated by sex. Shaded bands are 95% confidence intervals. WM growth is characterized by a concave function and a negative quadratic slope in both sexes, whereas BMI growth is characterized by a linear function with a slight positive quadratic slope in females.

The *χ*^2^ test revealed that constraining parameters to be equal between sexes produced a statistically significant increase in *χ*^2^, Δ*χ*^2^ (27) = 143.28, *p* < 0.001, suggesting that equality of parameters did not hold across gender. Iterative freeing of parameters suggested that the following parameters varied across sexes: the paths from Birthyear to the quadratic and the linear BMI slopes; the paths from Physical activity to the WM and BMI intercepts; the paths from Race/Ethnicity to the WM intercept and the quadratic BMI slope; and the paths from the WM intercept and the linear and quadratic BMI slopes. This last result suggests some sex-dependency in the way BMI and WM developmental trajectories are related. After these parameters were freed, there was no significant difference in *χ*^2^, Δ*χ*^2^ (19) = 30.12, *p* = 0.050. This partially constrained, multi-group model fitted the data well: CFI = 0.990; RMSEA = 0.039; SRMR = 0.017 (table 2). Variances of growth parameters for both WM and BMI were significantly different from zero in both sexes. The model-implied trajectories for WM and BMI are shown in figure 1. This model was based on data from 14 539 participants (7097 girls, 7442 boys).

**Table 2.** Goodness of fit indices and non-nested model comparisons for the conditional growth curve models. The winning model according to the AIC, a relative goodness of fit index, penalizing for model complexity, is highlighted in italics.

model	CFI	RMSEA	SRMR	AIC	*χ*^2^	d.f.
baseline	0.990	0.039	0.017	258173	1878	157
general adversity	0.989	0.039	0.019	149 468	1323	175
*dimensional adversity*	0.988	0.037	0.019	*149* *325*	1397	206

#### (ii) Identification of the WM and BMI latent growth curve parameters

We start with a description of the growth parameters and their associations.

*Linear slope parameters*. The estimated linear slopes of both WM and BMI were positive for boys (WM: *b* = 0.375, *p* < 0.001, 95% CI = [0.323, 0.426]; BMI: *b* = 0.193, *p* < 0.001, 95% CI = [0.154, 0.231]) and for girls (WM: *b* = 0.344, *p* < 0.001, 95% CI = [0.294, 0.394]; BMI: *b* = 0.188, *p* < 0.001, 95% CI = [0.151, 0.224]).

*Quadratic slope parameters*. The quadratic slope of the boy sample was negative for WM and did not significantly differ from 0 for BMI (WM: *b* = –0.029, *p* < 0.001, 95% CI = [−0.038, −0.020]; BMI: *b* = 0.000, *p* = 0.994, 95% CI = [−0.007, 0.007]). In the girl sample, the quadratic slope was negative for WM, and positive as trend for BMI (WM: *b* = −0.025, *p* < 0.001, 95% CI = [−0.034, −0.017]; BMI: *b* = 0.006, *p* = 0.074, 95% CI = [−0.001, 0.013]).

*Association between the intercept and the linear slope parameters*. The intercept and the linear slope significantly covaried in a negative way for WM and in a positive way for BMI, for both boys (WM: *b* = −0.103, *p* < 0.001, 95% CI = [−0.120, −0.086], *r* = −0.633; BMI: *b* = 0.058, *p* < 0.001, 95% CI = [0.053, 0.063], *r* = 0.412) and girls (WM: *b* = −0.075, *p* < 0.001, 95% CI = [−0.090, −0.060], *r* = −0.508; BMI: *b* = 0.057, *p* < 0.001, 95% CI = [0.052, 0.062], *r* = 0.465).

*Association between the intercept and the quadratic slope parameters*. The intercept and the quadratic slope significantly covaried in a positive way for WM and in a negative way for BMI, and so for both the boy sample (WM: *b* = 0.014, *p* < 0.001, 95% CI = [0.011, 0.017], *r* = 0.542; BMI: *b* = −0.003, *p* < 0.001, 95% CI = [−0.004, −0.002], *r* = –0.142) and the girl sample (WM: *b* = 0.009, *p* < 0.001, 95% CI = [0.007, 0.012], *r* = 0.399; BMI: *b* = −0.003, *p* < 0.001, 95% CI = [−0.004, −0.002], *r* = –0.160).

*Association between the linear and the quadratic slope parameters*. The linear and quadratic slopes covaried significantly and negatively for both WM and BMI, in boys (WM: *b* = −0.009, *p* < 0.001, 95% CI = [−0.011, −0.007], *r* = –0.924; BMI: *b* = −0.006, *p* < 0.001, 95% CI = [−0.007, −0.006], *r* = –0.725) so as in girls (WM: *b* = −0.008, *p* < 0.001, 95% CI = [−0.010, −0.006], *r* = –0.917; BMI: *b* = −0.004, *p* < 0.001, 95% CI = [−0.005, −0.004], *r* = –0.699).

The results reviewed so far indicate that for both sexes, the growth patterns of the WM and the BMI constructs in the measured time period are characterized by opposite acceleration effects. Whereas WM growth decelerates (positively signed linear slope parameter coupled with a negatively signed quadratic slope parameter), BMI growth shows a relatively stable linear increase with some evidence for acceleration in females (positively signed linear slope parameter coupled with a positively signed quadratic slope parameter).

#### (iii) Associations between the WM and BMI latent growth curve parameters

We now turn to interpret the estimated relationship between the growth curve parameters of the two constructs, and highlight sex differences, when appropriate. The WM intercept was negatively associated with the BMI intercept (*b* = −0.037, *p* = 0.001, 95% CI = [−0.059, −0.015], *β* = –0.043), and with the BMI linear slope (*b* = −0.032, *p* < 0.001, 95% CI = [−0.046, −0.018], *β* = –0.091). However, this latter finding emerged only in boys, as in girls no significant relationship emerged. This indicates that boys and girls who had relatively higher WM ability at age 6 also had relatively lower BMI at age 6, whereas boys who had relatively higher WM ability at age 6 exhibited a smaller degree of overall BMI growth between ages 6 and 11.

Instead, a relationship between the WM intercept and the BMI quadratic slope was found only in girls (*b* = −0.006, *p* < 0.001, 95% CI = [−0.009, −0.004], *β* = –0.137). This suggests that girls who had relatively higher WM ability at age 6 exhibited a smaller acceleration (or more deceleration) of BMI growth between ages 6 and 11.

Finally, for both girls and boys the WM quadratic slope was negatively associated with the BMI intercept (*b* = −1.982, *p* = 0.002, 95% CI = [−3.240, −0.724], *β* = –0.132) and the BMI quadratic slope (*b* = −0.123, *p* = 0.022, 95% CI = [−0.229, –0.017], *β* = –0.146). Hence, children of both sexes who had relatively higher acceleration (or less deceleration) in WM growth had a relatively lower BMI at age 6. They also exhibited a smaller acceleration (or more deceleration) of BMI growth. No other significant relationship was found between the growth curve parameters.

**Figure 2.**
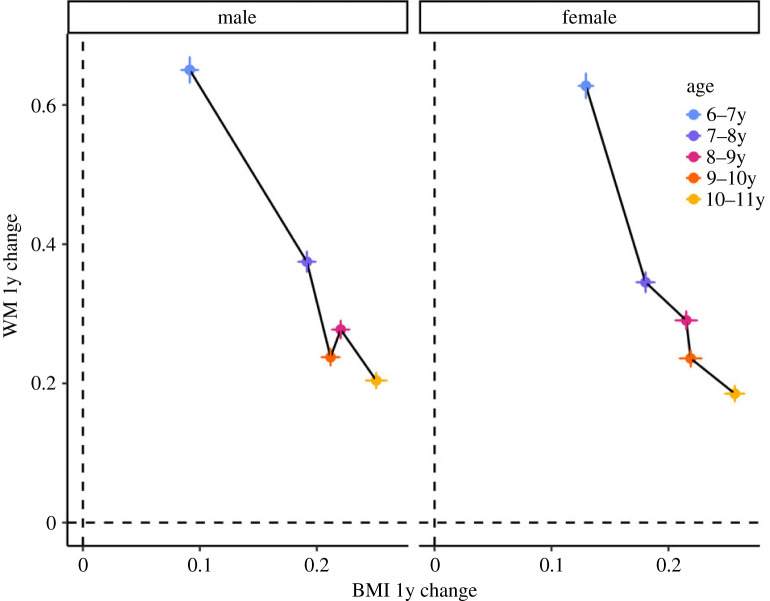
Yearly weight changes of BMI and WM, in the two sexes. Points are mean changes in WM and BMI standardized scores between successive years; error bars are 1 s.e. of the mean of these change scores. The negative relationship between WM and BMI velocities is indicated by the negative slope between the two variables, i.e. years that were characterized by relatively larger changes in BMI were characterized by relatively smaller changes in WM.

Overall, consistent with our *a priori* hypotheses and in line with the existing literature [6,12,45], our results imply a negative relationship between WM and BMI developmental trajectories assessed between ages 6 and 11, in both sexes. Figure 2 provides a visualization of this relationship by plotting change scores between time points on each construct, crudely approximating the derivative of WM and BMI growth curves. The pattern of associations between the WM and BMI growth parameters that we reported right above is coherent with the hypothesized energy trade-off between cognitive development and body growth, whereby a greater developmental rate of one system is counterbalanced by a smaller developmental rate of the other competing system. Here, a higher rate of cognitive development (indexed by WM ability) is counterbalanced by a lower rate of body growth (indexed by the BMI).

### (c) Conditional growth curves: general early life adversity model

While some recent papers have highlighted such a trade-off, particularly at an earlier period from birth to age 7, no study to our knowledge has hypothesized the role played by the quality of the environment in shaping its developmental dynamics. Therefore, we aimed to test whether the direct associations between the growth parameters remain consistent when the effect of early life adversity is taken into account. To this end, we added a composite score reflecting the general level of adversity experienced by children at age 6, as a predictor of the WM and BMI growth curve parameters. In this model, parameters that have been identified to vary across sexes in the baseline model described in the previous section were kept free. In this conditional growth curves model, we also tested for sex differences using a *χ*^2^ difference test approach.

#### (i) Goodness of fit

The *χ*^2^ test revealed that the partially constrained model fits the data equally well as the unconstrained model, Δ*χ*^2^ (25) = 33.157, *p* = 0.127, suggesting the equality of the additional adversity regression parameters across boys and girls. This partially constrained, multi-group model fitted the data well: CFI = 0.989; RMSEA = 0.039; SRMR = 0.019 (table 2). This model was based on data from 8673 participants (4208 girls, 4465 boys).

#### (ii) Effect of general early life adversity on the WM and BMI latent growth curve parameters

Adversity was positively associated with the BMI intercept (*b* = 0.005, *p* < 0.001, 95% CI = [0.003, 0.007], *β* = 0.057), but negatively associated with both the WM intercept (*b* = −0.017, *p* < 0.001, 95% CI = [−0.021, −0.014], *β* = −0.166) and the WM quadratic slope parameters (*b* = −0.001, *p* < 0.001, 95% CI = [−0.001, −0.001], *β* = −0.158). These results indicate that children who experienced relatively higher levels of general adversity at age 6 had higher BMI and lower WM at that age. They also experienced a more pronounced deceleration of WM growth between age 6 and age 11. Interestingly, adversity was also positively associated with the WM linear slope (*b* = 0.006, *p* < 0.001, 95% CI = [0.004, 0.008], *β* = 0.140), meaning that despite their lower initial levels of WM capacity, children experiencing greater adversity at age 6 exhibited an overall faster linear growth in their WM capacity.

Importantly, the inclusion of the general adversity composite in the model left the relationships between WM and BMI growth parameters largely unchanged, in terms of both direction and effect size. Indeed, the WM intercept still negatively and significantly predicted the BMI intercept in both sexes (*b* = −0.051, *p* < 0.001, 95% CI = [−0.079, −0.024], *β* = –0.059), the linear slopes in boys (*b* = −0.031, *p* < 0.001, 95% CI = [−0.048, −0.015], *β* = –0.092), and the quadratic slopes in girls (*b* = −0.005, *p* = 0.001, 95% CI = [−0.008, −0.002], *β* = –0.112). Similarly, the WM quadratic slope still negatively and significantly predicted the BMI intercept in both sexes (*b* = −1.976, *p* = 0.003, 95% CI = [−3.272, −0.680], *β* = −0.142). The only noticeable differences concerned the relationship between the WM and BMI quadratic slopes that now turned nonsignificant (*b* = −0.030, *p* = 0.584, 95% CI = [−0.138, 0.078], *β* = –0.039), and the relationship between the WM linear slope and the BMI intercept that now turned significant (*b* = −0.226, *p* = 0.025, 95% CI = [−0.424, −0.029], *β* = –0.102). Therefore, these results indicate that the negative relationship between WM and BMI development is not fully accounted for by the effects of early life adversity.

### (d) Conditional growth curves: dimensional early life adversity model

**Figure 3.**
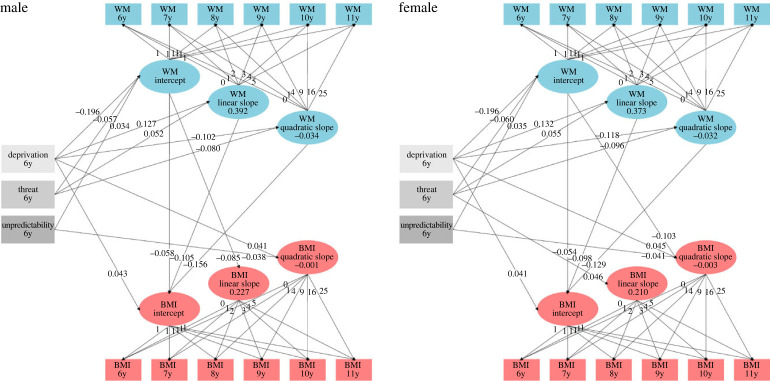
Simplified representations of the dimensional adversity male and female LGC models. Structures and standardized parameters of baseline model in males and females. Ellipses represent the growth parameters, modelled as latent variables, rectangles represent their indicators. The fixed loadings of indicators and significant regression paths are represented by bold lines and arrows. The mean unstandardized coefficients of the slope parameters are also indicated. In both sexes, deprivation and threat relate negatively to the WM intercept and quadratic slope, and positively to the WM linear slope. Deprivation also relates positively to the BMI intercept and quadratic slopes. Deprivation is also positively associated with the BMI intercept. Threat positively predicts the BMI linear slope only in women. Unpredictability, on the other hand, shows a positive relationship with WM intercepts and a negative one with BMI quadratic slopes.

Finally, we considered a more sophisticated model of adversity, in which the general adversity composite was split into three different dimensions known to have both shared and unique effects on psychological and physiological outcomes [15,29,46], and which reflect experiences of *Deprivation*, *Threat*, and *Unpredictability*. Scores on these three dimensions were then regressed on the growth curve parameters (figure 3).

#### (i) Goodness of fit

The *χ*^2^ test revealed that the partially constrained model fitted the data worse than the unconstrained model, Δ*χ*^2^ (37) = 55.353, *p* = 0.027, suggesting that the across-sex equality of the dimensional adversity regression parameters did not hold. Iterative freeing of parameters suggested that the following additional parameters varied across sexes: the path from Deprivation to the WM intercept; the paths from Threat to the linear and quadratic BMI slopes; the paths from Physical activity and Race/Ethnicity to the BMI linear slope. After these parameters were freed, there was no significant difference in *χ*^2^, Δ*χ*^2^ (32) = 43.828, *p* = 0.079. This partially constrained, multi-group model fitted the data well: CFI = 0.989; RMSEA = 0.039; SRMR = 0.019 (table 2). In fact, according to the AIC, a complexity penalized relative goodness of fit index, this dimensional model provided the best fit to the data. This model was based on data from 8673 participants (4208 girls, 4465 boys).

#### (ii) Effect of early life adversity dimensions on the WM and BMI latent growth curve parameters

*Deprivation*. First, deprivation was positively associated with the BMI intercept (*b* = 0.043, *p* = 0.001, 95% CI = [0.010, 0.040], *β* = 0.042) and the BMI quadratic slope (*b* = 0.001, *p* = 0.030, 95% CI = [0.000, 0.003], *β* = 0.041). Second, it was negatively associated with the WM intercept (*b* = −0.137, *p* < 0.001, 95% CI = [−0.162, −0.113], *β* = –0.196) and WM quadratic slope (*b* = −0.005, *p* < 0.001, 95% CI = [−0.007, −0.002], *β* = –0.111), but positively associated with the WM linear slope (*b* = 0.035, *p* < 0.001, 95% CI = [0.022, 0.048], *β* = 0.130).

*Threat*. Threat showed a similar pattern of associations: a negative association with both the WM intercept (*b* = −0.040, *p* < 0.001, 95% CI = [−0.060, −0.020], *β* = –0.059) and the WM quadratic slope (*b* = −0.004, *p* = 0.001, 95% CI = [–0.006, 0.002], *β* = –0.088), paralleled by a positive association with the WM linear slope (*b* = 0.014, *p* = 0.025, 95% CI = [0.002, 0.027], *β* = 0.054). Note that in girls only, Threat was found to be positively linked with the BMI linear slope (*b* = 0.010, *p* = 0.036, 95% CI = [0.019, 0.048], *β* = 0.046).

*Unpredictability*. Finally, unpredictability had a quite distinct pattern of associations. First, unpredictability had a specific negative effect on the BMI quadratic slope (*b* = −0.001, *p* = 0.024, 95% CI = [−0.002, 0.000], *β* = –0.040), and a positive effect on the WM intercept (*b* = 0.024, *p* = 0.016, 95% CI = [0.004, 0.043], *β* = 0.035).

It is noteworthy that the effects of the WM growth parameters on the BMI growth parameters remained largely unchanged from the general adversity model. Overall, adversity showed generally positive associations with BMI, and negative associations with WM, suggesting that more adverse backgrounds can contribute to increase energy expenditure on somatic development, as opposed to brain development (the current results do not tell us whether this contribution is direct or indirect). Further, our results suggest that different dimensions of adversity have partially distinct effects on WM and BMI development. Deprivation seemed the most strongly related to both WM and BMI in both girls and boys. Threat showed similar albeit weaker and less consistent effects. Unpredictability was uniquely associated with the deceleration of BMI growth and with somewhat increased WM ability at age 6. Interestingly, our results also imply that despite lower initial levels, children from more adverse conditions exhibited faster overall growth of WM ability.

## 4. Discussion

Previous work has generally found negative relationships between EF (and its neural correlates in the frontal lobe) and BMI, at multiple developmental stages [25,45,47], such that increasing body mass links with decreasing executive function ability. It has been proposed that this negative coupling could partially result from energy allocation decisions made by the organism during development, balancing energy expenditure towards somatic investment and brain development in an optimal fashion [1,6,8,48]. Importantly, this body–brain energy trade-off is subject to large between-individuals variations, whose sources remain largely unexplored. Better identifying these sources of variations could provide a more precise mechanistic explanation of the correlations often reported in the medical literature between patterns of somatic and neurocognitive growth and physical and mental health problems [14,49]. In this respect, it is surprising to see in the existing literature the relative lack of accounting for environmental factors that might influence this trade-off [22,50,51]. In this study, we hoped to make progress towards filling this gap, by estimating the association between trajectories of neurocognitive (measured by WM ability) and somatic (measured by BMI) development, while taking into account multiple dimensions of early life adversity. Several lines of results emerged. Our initial growth curves model revealed a negative coupling between initial WM ability and BMI values in both girls and boys of the sample, paralleled by a negative coupling between initial WM ability and BMI linear trajectories in boys and BMI quadratic trajectories in girls. In a subsequent model, we showed that early life adversity had positive associations with BMI trajectories and negative associations with WM trajectories, although the direct negative coupling between them remained consistent. Finally, our multidimensional adversity model revealed that it is the dimension of Deprivation, reflecting lack of access to resources and cognitive stimulation, that contributed the most to the WM and the BMI growth patterns.

The specific effect of Deprivation on WM is not unexpected. Prevailing dimensional models of adversity propose that Deprivation has a relatively specific detrimental effect on fronto-parietal neural circuitry underlying linguistic processes and executive functions [15,46]. This also echoes findings from animal studies, showing maternal deprivation leading to both cognitive deficits and neurobiological changes, most consistently in the hippocampus and the prefrontal cortex [52–55]. More interesting is the prevailing effect of this dimension on somatic development, with a positive association with both initial BMI (measured at age 6) and on the BMI quadratic growth term. It implies that exposure to deprivation can contribute to overweight and obesity both directly through increased somatic growth and indirectly through reduced EF development. These combined effects could reflect a life history strategy characterized by an increased investment into somatic growth and a decreased investment into neurocognitive growth, as an adaptive response to specific forms of early life adversity.

Our models also revealed some surprising associations between WM and adversity dimensions. The first of which is the fact that children more severely exposed to Deprivation and Threat exhibited a larger degree of linear growth WM ability between ages 6 and 11, despite lower initial levels at age 6. This suggests that there is a certain degree of ‘catch up’ in such children. Moreover, Unpredictability had a small, but positive association with initial WM ability, suggesting that not all forms of adversity have the same association with neurocognitive development. These results might be interpretable as evidence for so-called ‘hidden talents’. A number of studies support the view that certain forms of adversity can lead to enhanced cognitive functions, such as task switching, WM, and some forms of social information processing [56–59]. While most of this work has been in adults, there is preliminary evidence of such cognitive enhancements already emerging in children. A recent, large study has found unpredictability to be uniquely linked with increased performance on a WM test, in adolescent children [60]. A study among Nigerian youth has also revealed improved WM ability in deprivation-exposed children, compared to non-deprived controls [61].

To the best of our knowledge, no study so far has investigated the link between neurocognitive and somatic growth, and incorporated a thorough, dimensional characterization of early adversity. We believe our work adds to the existing literature in multiple ways. Firstly, by replicating earlier work, showing a negative coupling between EF and BMI growth during childhood [12,25], within the normal BMI range, lending support to the theory of Kuzawa & Blair [6,12] regarding the brain–body energetic trade-off. Secondly, we also extend this line of research. On the methodological side, our study benefits from: (i) a larger number of timepoints, allowing for more complete growth curves; (ii) a large and representative sample of children, leading to strong generalizability; (iii) reliable WM assessment using a valid and reliable experimental task and during an age when psychometric assessments of EF are robust [62]; and (iv) incorporation of important confounds, such as physical activity, gender, ethnicity, and most crucially, early life adversity. On the conceptual side, we couple the estimation of EF and BMI growth curves and their interrelationship with a thorough, dimensional assessment of early life adversity, allowing for more specificity in examining its role [26]. Indeed, this conceptual split of the adversity construct allowed us to reveal rather distinct effects of different dimensions, with Deprivation being especially predictive of both increased somatic and decreased neurocognitive growth. This has important implications for interventions and casts a new light on reports of the effects of early educational programmes on physical health (e.g. [63]). The surprising small, positive impact of Unpredictability of WM ability is also noteworthy, and its potential explanation in terms of ‘hidden talents’ is an intriguing possibility that is worth exploring for future research [60,63]. None of these associations would have been uncovered had we relied on general adversity composite scores.

Notwithstanding, our study suffers from several limitations as well. Firstly, although the relatively later developmental period we targeted here has likely contributed to an accurate measurement of EF, it also is notably later than the period when the steepest brain–body trade-offs are hypothesized to take place [1]. Relatedly, we lack any information regarding the status of our participants at these earlier ages, with respect to adversity, neurocognitive or somatic growth. All these earlier effects are thus unaccounted for in our modelling. We similarly lacked information about participants' subjective perceptions regarding the effect of exposure to the experiences that we labelled as ‘adverse’. This is a crucial shortcoming, as a number of studies have highlighted that the subjective impact of an adverse event is often more important than mere objective occurrence in predicting mental and physical health outcomes [64–68]. We also note that our Threat composite largely captured extrafamilial and neighbourhood level threat, which is only one part of the dimension. It remains for future studies to extend our results by a wider ranging operationalization of this construct. Finally, our use of BMI and WM ability as proxies of bodily and brain energy expenditure is of course indirect, and much caution has to be taken with interpretation. As BMI is calculated using total body mass, it contains two factors that have distinct biological effects, adipose tissue and lean mass [69]. Moreover, the correlation between BMI and these two distinct sources of mass appears to be different in men and women to some degree [69], which might also explain the relative lack of sex differences in somatic growth in our study. Other physical growth parameters, such a waist-to-hip ratios, should also be taken into account when characterizing energy allocation [51]. Similarly, the relation between EF ability and brain energy use is far from clear, with some studies finding negative, instead of positive correlations between brain energy use and cognitive ability [70,71]. Nevertheless, BMI has been shown to correlate strongly and linearly with more precise estimates of fat mass in a wide range of studied populations, with correlations ranging from 0.67 to as high as 0.95 (reviewed in [13]). Furthermore, BMI is a known risk factor for metabolic and cardiovascular disease [72,73], and is a component of the so-called metabolic syndrome (which includes an overweight component), highlighting its close relationship with the body's (sometimes maladaptive) energy allocation [74]. Similarly, studies across the lifespan show that peak brain energy demands coincide both with the developmental stage with the sharpest EF improvements [10,75,76], and with the nadir of weight gain [1,6,7,9]. A landmark study by Goyal and colleagues [7] further revealed adult prefrontal regions having the highest levels of aerobic glycolysis that also correlate with the persistence of brain maturational changes. It thus seems that a large fraction of both childhood and adult brain energy is expended on the fine tuning of neural networks by continued synaptic plasticity (especially in prefrontal cortex) that is necessary for complex cognitive functions (especially EF). All of this suggests that BMI and EF ability growth curves are acceptable, although imperfect proxies of energetic investment into somatic and neurocognitive growth.

Overall, we believe our study improves our understanding of an important public health issue, and provides an integrative, evolutionary-developmental framework, within which to interpret the complex interrelationships between early life adversity, neurocognitive development and physical growth.

## Data Availability

All pre-processed data and code necessary to reproduce all results in the paper are available at the OSF framework (https://osf.io/ezscm/). The raw data are similarly available after the appropriate steps from the ECLS-K:2011 study website (https://nces.ed.gov/ecls/kindergarten2011.asp). Supplementary material is available online [77].
